# Phthalate exposure among U.S. college-aged women: Biomonitoring in an undergraduate student cohort (2016-2017) and trends from the National Health and Examination Survey (NHANES, 2005-2016)

**DOI:** 10.1371/journal.pone.0263578

**Published:** 2022-02-11

**Authors:** Barbara A. Beckingham, Kerry Wischusen, Joanna P. Walker

**Affiliations:** 1 Department of Geology & Environmental Geosciences, College of Charleston, Charleston, South Carolina, United States of America; 2 Department of Biochemistry, College of Charleston, Charleston, South Carolina, United States of America; 3 Department of Health and Human Performance, College of Charleston, Charleston, South Carolina, United States of America; Shahjalal University of Science and Technology, BANGLADESH

## Abstract

**Importance:**

Phthalates are ubiquitous and many are known or suspected human reproductive and endocrine-disrupting toxicants. A data gap exists in reporting on biomonitoring of phthalate biomarkers in college-aged adults.

**Objective:**

To analyze phthalate exposure in a cross-sectional sample of female college students using urinary phthalate metabolite concentrations and compare to reference populations including college-aged women sampled in the National Health and Nutrition Examination Survey (NHANES).

**Methods:**

Nine monoester phthalate metabolites were analyzed in spot urine collected from 215 female undergraduates (age 18–22, 2016–2017) at a public university in Charleston, SC USA and a subset of participants completed a questionnaire detailing demographics and behaviors including personal care and cosmetic product use (e.g. in the past 6 or 24 hrs). Urine specific gravity was used to assess effect of urine dilution. Phthalate metabolite concentrations were compared to reference populations and the temporal trends of the same age-group in the National Health and Nutrition Examination Survey (NHANES) were analyzed.

**Results:**

Total urinary phthalate metabolite concentrations in individuals ranged three orders of magnitude (geometric mean 56.6 ng/mL, IQR 26.6–114 ng/mL). A third of urine samples had relatively high urine specific gravity levels indicating potential dehydration status. All geometric mean concentrations were similar to the U.S. female population in the most recent NHANES cycle (2015–2016) except for MEP and mono-isobutyl phthalate (MiBP). Relatively low MEP and MiBP may be explained by a time trend of declining MEP in the general U.S. population, the sociocultural character of this cohort, and the time of day of spot sampling in evening. NHANES data indicate a significant effect of sample timing on phthalate metabolite concentrations and decline in most, but not all, phthalate metabolites sampled in women aged 18–22 years over the decade (2005–2016).

**Significance:**

This study reports phthalate metabolites in college-aged women, an understudied group, emphasizes the benefit of survey information for interpreting biomonitoring data, and is a useful case study for communicating phthalate chemical exposure risks to college students.

## Introduction

Phthalates are a group of phthalic acid diesters that are high-production volume chemicals used in a wide range of products, from personal care products, plastics (e.g. PVC), adhesives and sealants to pesticides. Given the ubiquity of phthalates, exposure pathways are both direct (e.g. contact with personal care products and plastics or medical equipment) and indirect (e.g. uptake from food or dust), and occur through oral, inhalation or dermal routes depending on the properties and uses of different phthalate chemicals [1, 2]. Human health concerns stem from their endocrine-disrupting activity and associations with adverse reproductive system outcomes [3–5]. Previous studies have also shown associations between phthalate exposure and symptoms of depression, anxiety and stress in young adults [6]. Phthalates in humans are metabolized to phthalate monoesters or other oxidative metabolites and glucuronide conjugates that are more readily eliminated in urine [7]. Urinary phthalate metabolite concentrations are therefore used extensively as exposure biomarkers.

Since half-lives of phthalates in the body are on the order of <24 hrs, their presence in urine reflects short-term exposure behaviors [3, 8, 9]. Even so, studies have shown that a single spot urine sample can reasonably classify phthalate exposure through the detection of phthalate metabolites within a monthly or seasonal timeframe [9, 10] since, due to their widespread use, phthalate exposure is relatively routine and continuous. Certain phthalate metabolites may be more variable than others due to their biotransformation rate or link to more episodic exposure routes [11, 12]. Sampling conditions (e.g. collection time, frequency, and integration) are important to consider to correctly classify exposure in biomonitoring studies [12, 13].

Researchers have previously reported on phthalate metabolites detected in female populations in the United States, particularly during pregnancy [8, 14–17] or in cohorts of reproductive age [18], or adolescent age specifically [19]. Globally, college students have been assessed for phthalate exposure in studies in Germany (ages 19–29 years) [20, 21] and China (ages 17–24 years) [6]. The Centers for Disease Control (CDC) analyzed levels of phthalate metabolites in urine in the United States population in the National Health and Nutrition Exaination Survey (NHANES) between 1999 and 2016 [22–24]. NHANES surveys young adults of traditional college age and collects demographic information including educational attainment, but to date, this population has not been a focus. Therefore, there is a data gap in biomonitoring of endocrine disrupting chemicals, and specifically phthalate biomarkers, in college-aged young adults.

Given the frequent use of products that may contain phthalates (e.g. beauty and personal care products) [25] and likely preference for convenience to combat busy academic schedules (e.g. consumption of “fast foods” and use of plastics in food preparation and consumption), we hypothesized that college females would be particularly susceptible to phthalate exposure. This study reports on the levels of nine phthalate metabolites in urine from a cross-sectional sample of a college female student population in Charleston, SC USA. Traditional post-secondary students in the United States (e.g. students enrolled full-time within a year of graduating high school) are most likely to be 18–22 years old and for simplicity are referred to in this paper as “college-aged”, although we recognize that people of all ages attend university. Phthalate metabolite concentrations are compared to reference populations, including 18–22 years old females surveyed in NHANES over time (2005–2016), and the effect of sampling conditions and exposure-linked cosmetic and personal care product use is assessed to interpret biomonitoring results.

## Material and methods

### Sample and survey collection

**Fig 1:**
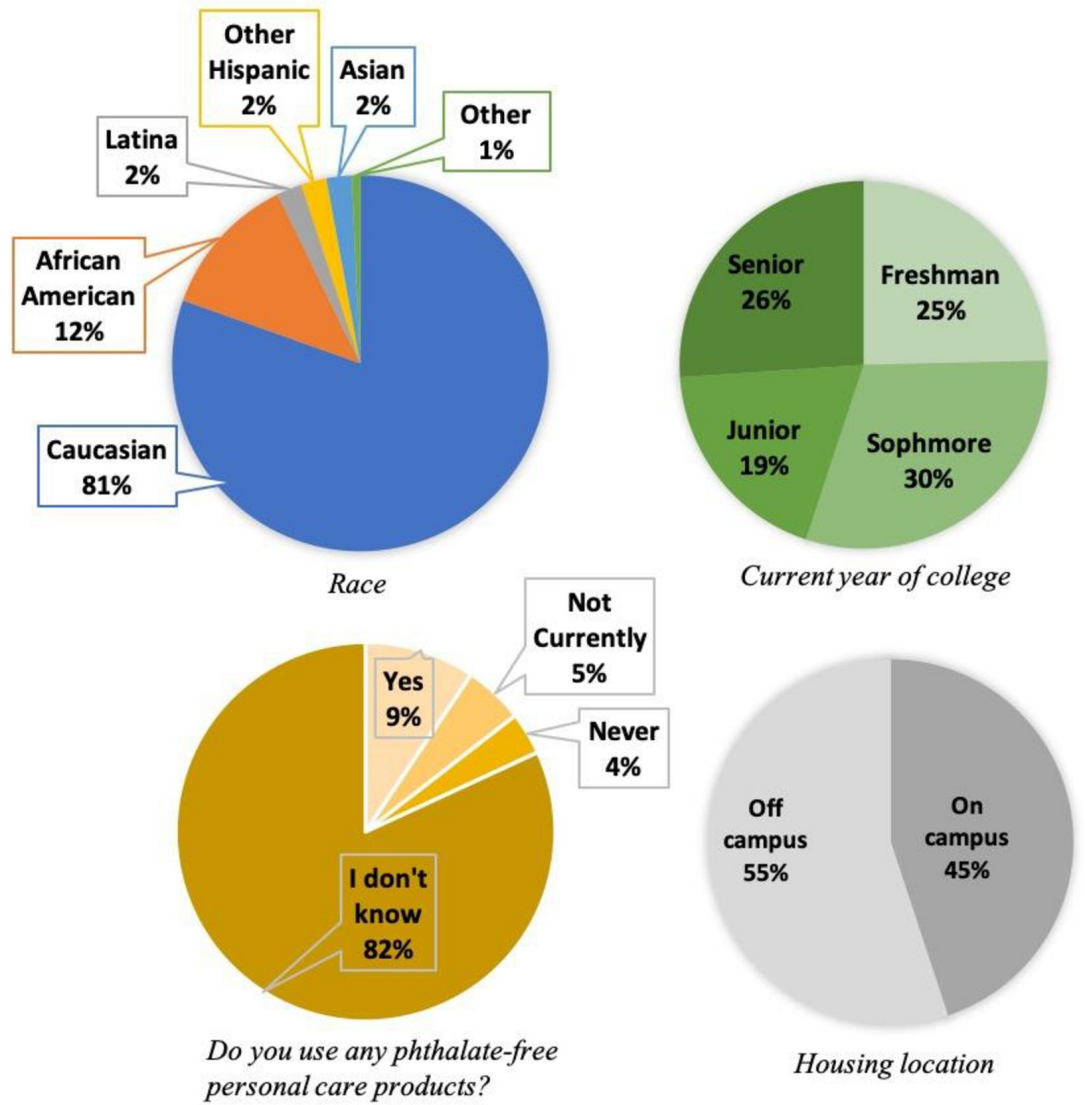
Reported personal information from surveyed undergraduate students. Additional characteristics (parental education, alcohol and tobacco use and personal care and cosmetic use) are tabulated in Hart et al. [25].

Female undergraduate students (age 18–22 years, non-pregnant) at a public university in Charleston, SC USA were recruited to participate in this study during the academic year. Study participants came to one of several open clinics held during weekday late-afternoon/evenings (4–6 pm, Mondays-Thursdays) between November 2016 and February 2017. Participants provided a spot urine sample in a sterile plastic specimen container that was measured for specific gravity (clinical hand-held refractometer, Fisher Scientific) then immediately frozen and stored at -4°C until sample processing for stability of the sample [26]. Participants also completed a self-administered survey that included questions pertaining to demographic and socioeconomic characteristics in addition to behavioral information on residence, and use of smoking products, cosmetics, personal care products and plastic food containers as described and reported in Hart et al. [25]. A subset of this information (race, housing and year of study) is displayed in Fig 1. Phthalate-free products were offered as incentives for participation and all participants provided informed consent for this institutional review board-approved study.

### Phthalate metabolite measurements

Nine phthalate monoester metabolites were measured in urine following protocols for sample processing and analysis in previous biomonitoring studies [17, 27]: monomethyl phthalate (MMP), monoethyl phthalate (MEP), monobutyl phthalate (MBP), mono-isobutyl phthalate (MiBP), monobenzyl phthalate (MBzP), mono(2-ethylhexyl) phthalate (MEHP), mono(2-ethyl-5-oxohexyl) phthalate (MEOHP), mono(2-ethyl-5-hydroxyhexyl) phthalate (MEHHP) and mono-isononyl phthalate (MNP). In brief, urine samples were thawed and vortexed, then a 1 mL aliquot of urine was spiked with deuterated phthalate monoester internal standards, subjected to enzymatic deglucuronidation followed by isolation on solid phase extraction cartridges and subsequent elution. Each sample was then transferred to deionized water for analysis by high-performance liquid chromatography with electrospray ionization tandem mass spectroscopy (LC-ESI-MS/MS). All materials for sample preparation and instrument parameters for detection were reported previously [28]. Matched ^13^C-labelled phthalate metabolite internal standards are listed in S1 Table.

Quality assurance/quality control samples processed along with urine samples and calibration standards included DI blanks prepared in the laboratory (N = 20), urine sample duplicates (N = 17) and matrix spikes with each sample batch (N = 10), specimen container blanks prepared with DI water that was stored frozen in specimen containers (N = 2), and National Institute of Standards and Technology Standard Reference Material (NIST, SRM 3673 Organic Contaminants in Non-Smokers’ Urine (Frozen), N = 5). Limit of detection (LOD) for each analyte was determined based on a method correlating standard deviation of replicate standard measurements with concentration [29] and ranged from 0.1 ng/mL to 2.4 ng/mL for individual phthalate monoesters (LOD of present and reference studies is provided in S1 Table).

### Data and statistical analysis

Phthalate metabolite concentrations in spot urine samples from reference populations analyzed by the same analytical methods were obtained from the peer-reviewed literature [17] and from the CDC NHANES database for cycles 2005–2006 to 2015–2016 (cdc.gov/nchs/nhanes). Phthalate metabolites were not included in the laboratory variables measured in urine for NHANES 2017–2018. Additional survey information of participants in NHANES (e.g. age, sex, education, race/ethnicity, sample timing) was also acquired from the database and cross-referenced to urinalysis results. NHANES participants age 18–22 years are identified as the “college-aged” sample, regardless of educational attainment (sample sizes are reported in S3 Table). Reported education and race of NHANES participants in the 2013–2014 and 2015–2016 cycles are summarized in S4 Table. In analysis of NHANES biomonitoring results over time, phthalate metabolite concentrations are reported as adjusted concentrations with weighting factors applied provided by the NHANES database for the urinalysis subpopulation to account for differences in demographics in comparison to the U.S. civilian population in each cycle over time, as recommended by the CDC. Raw data was analyzed from the cosmetic and personal care product use survey reported in Hart et al. [25], and while we analyzed urine of 215 participants, only 133 participants matched to a completed survey.

Statistical analyses were performed in Microsoft Excel (V.16.32) and JMP Pro (V.12.0, V.15.1). Peaks quantified <LOD were included in statistical analyses as LOD/sqrt2. If >60% of samples were quantified <LOD, geometric means are calculated but flagged [22]. Phthalate metabolite concentration data met assumptions of equal variance according to Levene’s test and were log-transformed to meet assumptions of normality for statistical tests with significance set at *α* = 0.05. Specific-gravity adjusted concentrations (C_S.G.,_ ng/mL) were calculated using the formula C_S.G._ = C_a_((S.G._avg_− 1)/S.G._a_− 1)) where C_a_ and S.G._a_ are the phthalate metabolite concentration and specific gravity of an individual urine sample, respectively, and S.G._avg_ is the average specific gravity of the entire sample set.

## Results and discussion

### Quality assurance/control

Analytical methods used for urinalysis passed quality assurance/control checks. Reproducibility of sample duplicates and recovery of matrix spike and SRM expected concentrations were acceptable. Specifically, all compound matrix spike recoveries were above 70% on average and SRM results compared within +/- 40% of expected values (S2 Table). Matrix spike recovery did not correlate to urine specific gravity. There were no phthalate metabolites detected associated with plastic containers used to store urine. All deionized water blanks were clear of phthalate metabolite peaks with the exception of discrete occasions of MEP (N = 8, all <LOD), MBP (N = 7, 4>LOD) and MiBP (N = 4, 2>LOD). Average concentration values in blanks were subtracted from sample data for blank correction on these batch quantification dates.

### Phthalate metabolites urinalysis

#### Urine specific gravity

Adjustment of sample concentration to specific gravity or creatinine may be made to normalize for the effects of urine dilution due to variation in hydration and excretion among participants of a study. Average urine specific gravity measured in the present study was 1.024 ± 0.012 (ranged 1.022–1.052; N = 215), which is high in comparison to reference populations. For instance, average S.G. of 1.014 and 1.016 were reported among pregnant females enrolled in biomonitoring studies also in Charleston, SC [16, 17, respectively]. However, other studies have reported on urine samples with relatively high specific gravity. A workplace study (N = 294) reported ~50% of samples with S.G. >1.020, and several (N = 5) >1.040 [30]. Some researchers suggest a specific gravity cut-off above which samples are removed from a data set. For example, the U.S. federal policy for workplace testing mandates an acceptable specimen to range between 1.001 and 1.02 [31], while intergovernmental authorities of industrial hygienists extend the acceptable range to 1.03 [32]. An occupational database study of N = 2385 found 4% of adult female samples to be concentrated above S.G. 1.03 [32]; we find 36% of samples above 1.03. Given these previous recommendations, the data set is analyzed in its entirety and as 1.03 > S.G. ≥1.03. Relatively high urine specific gravity measured for female undergraduate students may indicate an issue with dehydration. While adults with adequate fluid intake and normally functioning kidneys can produce urine with a wide range of specific gravity (e.g. 1.003–1.035) [33], specific gravity above 1.03 likely indicates dehydration [34].

#### Phthalate metabolite concentrations and comparison to reference populations

Urinary phthalate metabolite concentrations in this female undergraduate cohort are summarized in Table 1 (full dataset provided in S2 File). MEP was the phthalate metabolite most often detected at the highest concentrations on average, which is consistent with previous studies [e.g. 17, 22]. MBzP, MiBP, MBP, MEHHP and MEOHP were also ubiquitous (quantified in >99% and >LOD in 92–100% of samples). Frequency of detection (>LOD) of MEHP (40%) and MMP (21%) was lower than some other studies [e.g. 17]. MNP was not present or <LOD in all samples, and it is also rarely detected in nation-wide surveys [22]. The highest concentration detected was for MBP in one sample (11300 ng/mL), and this extreme value was censored and excluded in further analysis. The sum of phthalate metabolites in samples ranged three orders of magnitude, from 1.9 ng/mL to 1342 ng/mL. Geometric mean concentrations of each phthalate metabolite for samples with S.G. above 1.03 (N = 77) were elevated in comparison to samples below 1.03 (N = 138), as shown in Table 1, as would be expected as a function of urine dilution.

**Table 1:** Summary of measured urinary phthalate metabolite concentrations. Geometric mean^1^, median (and interquartile range) concentrations (ng/mL) and number [N] above and below specific gravity (S.G.) of 1.03 in addition to unadjusted and S.G.-adjusted (using sample mean S.G.) concentrations for the entire dataset. Maximum concentrations detected in the entire dataset (all data) [N = 215] are also given^2^.

Compound	S.G.<1.03	S.G.≥1.03	All data-unadjusted	All data-S.G.-adjusted
MBP ^3^	6.7	20.6	9.7	11.2
	6.7 (3.8–12.8)	20.3 (12.0–33.4)	10.3 (5.2–20.1)	11.3 (7.1–16.8)
	[136]	[77]	[213]	[213]
			Max 83.2	Max 81.1
MBzP	2.9	7.7	4.1	4.8
	3.1 (1.1–6.1)	7.7 (4.7–14.4)	4.7 (2.2–9.4)	5.1 (2.8–7.7)
	[136]	[77]	[213]	[213]
			Max 230	Max 131
MEHHP	1.4	2.4	5.7	6.7
	(0.6–2.3)	(1.3–4.9)	5.9 (2.9–11.1)	6.5 (4.3–10.5)
	[138]	[77]	[215]	[215]
			Max 200	Max 117
MEHP	1.2	2.3	1.8	1.4
	0 (0–0.6)	1.7 (0.6–3.2)	0.6 (0–1.7)	0.4 (0–1.2)
	[47]	[62]	[109]	[109]
			Max 44.6	Max 26.1
MEOHP	2.4	7.6	3.6	4.3
	2.5 (1.3–4.5)	7.0 (4.2–13.4)	3.7 (1.8–7.0)	4.1 (2.7–6.8)
	[138]	[77]	[215]	[215]
			Max 134	Max 78.4
MEP	11.0	30.2	15.8	18.5
	10.0 (4.2–25.3)	28.8 (13.6–54.8)	16.2 (6.0–38.4)	15.5 (8.0–33.6)
	[138]	[77]	[215]	[215]
			Max 1290	Max 1238
MiBP	3.8	12.4	5.8	6.7
	3.9 (1.7–7.6)	12.6 (7.7–18.3)	6.3 (2.9–21.1)	7.0 (3.7–10.9)
	[137]	[77]	[214]	[214]
			Max 127	Max 58.6
MMP	1.1	1.8	1.4	1.6
	0.8 (0–0.8)	0.8 (0–1.4)	0.8 (0–0.8)	0.5 (0–1.6)
	[137]	[45]	[116]	[116]
			Max 56.7	Max 41.2
Total	38.5	113	56.6	66.4
	37.9 (20.5–72.0)	101 (76.0–156)	62.0 (26.6–114)	61.3 (42.7–97.9)
			Max 1342	Max 1288

^1^ Underlined values fail the criteria of %samples >LOD of >40% for calculation of geometric mean [22]. Geometric mean calculation includes samples <LOD as LOD/sqrt2.

^2^ Minimum concentration was either 0 (non-detect) or LOD/sqrt2 for all metabolites.

^3^ Calculation of geometric mean and maximum values does not include 1 extreme value of 11300 ng/mL.

Geometric mean unadjusted concentrations are compared to other studies in Table 2. Unadjusted concentrations were used since it is common for studies to use creatinine instead of specific gravity to correct for urine dilution (e.g. NHANES), or to not report adjusted values. Phthalate metabolite concentrations in the present study are lower than reported in Wenzel et al., who measured phthalate metabolites in a racially and socioeconomically diverse population of pregnant females in Charleston, SC USA from 2011 to 2014 [17]. This comparison is likely affected by the year of sampling and the demographic of study participants. Fifty percent of participants in Wenzel et al. identified as African American and the other 50% as Caucasian/white, and the median age (interquartile range, IQR) was 27 (8) years old [17]. Wenzel et al. found a statistically significantly higher phthalate exposure for African-American women that may have been the result of differences in metabolism or exposures (e.g. personal care product use and diet), and additionally found that college-educated women had lower urinary phthalate metabolite concentrations [17]. In contrast, the study population in the present study is comprised of 12% African-American students and 80% Caucasian/non-hispanic white students, which is fairly representative of the college student body from which the sample was drawn. Race and socioeconomic factors have been shown to affect exposure to phthalates among women of reproductive age in a study using pooled NHANES data from 2001–2008, but race was found to be a factor only for MEP, MBP/MiBP and MBzP [18]. Specifically, MEP was found to be 0.75-times lower for non-hispanic whites, leading the authors to “cautiously” interpret this as a sociocultural difference in personal care product use. Non-white racial groups also showed higher concentrations of MBP/MiBP in this NHANES study [18]. Sociocultural and socioeconomic factors can also influence dietary phthalate exposure, as reviewed previously [35]. Exposure to many phthalate chemicals in the U.S. population has also been declining in recent years. Median concentrations of urinary phthalate metabolites among Americans, and specifically women, have declined over the period 2000 to 2014 [22, 36]. Phthalate metabolite concentrations detected in college females are similar to the NHANES 2015–2016 survey data reported in Table 2 for all U.S. females sampled (ages 3–80) [22] and college-aged females in NHANES 2013–2016, likely due to these sampling years being more proximate in time. The exceptions were MEP and MiBP, for which 95% CI do not overlap and geometric mean unadjusted concentrations were 0.43 and 0.71 times those reported for NHANES 2015–2016 females, respectively.

**Table 2:** Geometric mean unadjusted concentrations (ng/mL) with 95% confidence intervals (CI) of urinary phthalate metabolites in comparison to reference populations.

Compound^1^	Present study [N = 215]	95% CI	Wenzel et al. 2018 [17] [N = 378]	95% CI	NHANES 2015–16 all females^3^ [N = 1501]	95% CI	NHANES 2013–16 college-aged females^4^ [N = 184]	95% CI
MBP	9.7^2^	8.5–11.1	13.7	12.1–15.5	9.78	8.77–10.9	11.6	10.1–13.3
MBzP	4.1	3.5–4.8	9.47	8.06–11.1	4.35	3.76–5.03	6.99	5.92–8.26
MEHHP	5.7	5.0–6.5	6.34	5.70–7.10	5.27	4.89–5.66	7.30	6.14–8.67
MEHP	1.8		2.65	2.36–2.99	x		x	
MEOHP	3.6	3.2–4.1	5.02	4.50–5.62	3.42	3.14–3.72	5.11	4.32–6.04
MEP	15.8	13.1–19.0	47.0	39.3–55.5	36.5	30.2–44.0	45.1	37.2–54.7
MiBP	5.8	5.0–6.7	9.57	8.44–10.8	8.16	7.31–9.12	10.9	9.14–12.7
MMP	1.4		1.92	1.66–2.23	x		x	

^1^ MNP is not included in this table since this analyte was not present or <LOD in all samples; MNP was not measured in Wenzel et al. 2018 [17] and was not reported in NHANES 2015–2016.

^2^ Excluding extreme value of 12300 ng/mL in 1 individual (geometric mean is 10.1 ng/mL with this value included).

^3^ Weighted values reported by CDC [22].

^4^ Weighted values to scale the sample in alignment with U.S. civilian population demographics are calculated for the college-aged (18–22 years) females subset of combined NHANES 2013–2014 [N = 116] and 2015–2016 [N = 68] cycles. Education and race data for this group are tabulated in S4 Table and data by sampling time in S1 File.

x: Not reported by CDC [22] because >40% of samples <LOD. Underlined values have >40% of samples <LOD. 95% CI is not determined.

**Fig 2:**
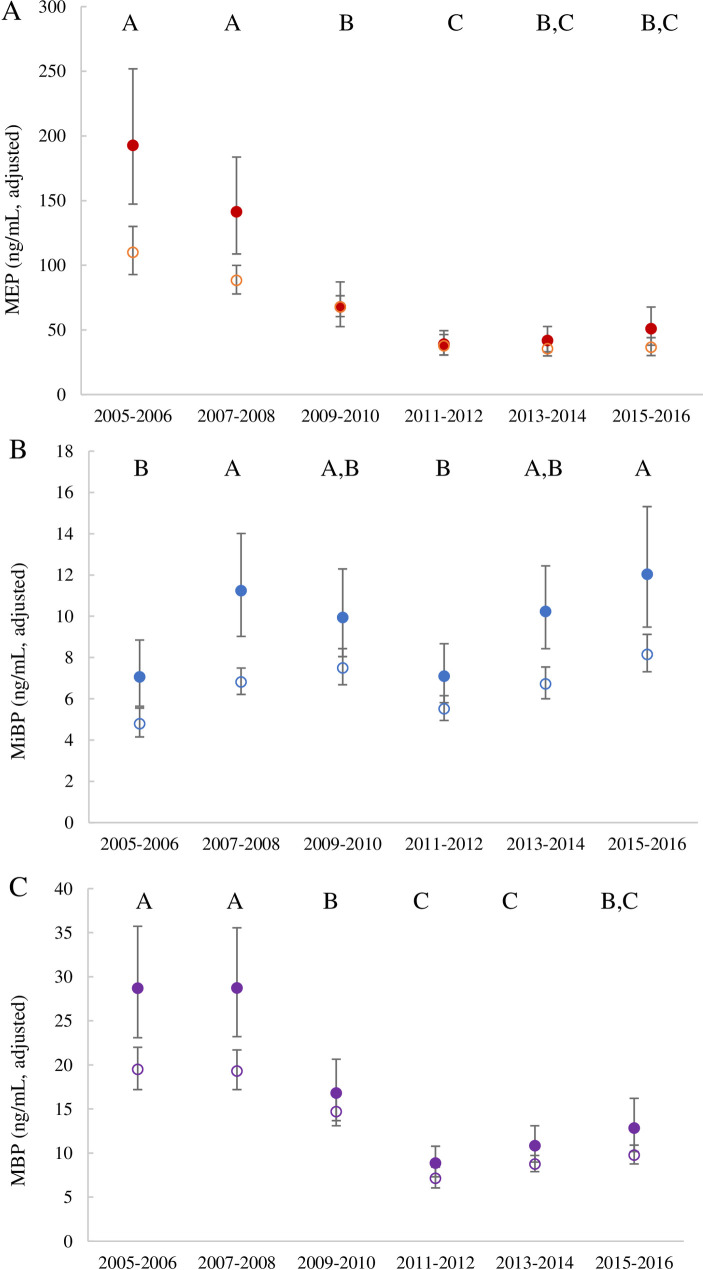
Geometric mean adjusted concentrations for college-aged (18–22 years old) females (filled circles) and for all females (open circles) in NHANES reported in the 4^th^ National Assessment [22] over time for A) MEP, B) MiBP and C) MBP. Bars indicate 95% confidence intervals. Note, not all data are for women with college educations. See S3 Table for sample sizes over time. Years when college-aged females have detectably different phthalate metabolite concentrations using log-transformed adjusted concentrations by ANOVA with Tukey’s significance test (p<0.05) are shown with different letters in each panel.

College-aged students sampled as part of NHANES have total phthalate metabolite urinary concentrations that are statistically significantly lower in years after 2009 compared to previous years (One-way ANOVA with Tukey’s significance test, p<0.001; S1 Fig). Trends for the college-aged female subpopulation follow and extend those shown for the wider NHANES populations from 2001–2010 [24]. Therefore, comparisons to studies conducted prior to 2010, before concentrations may have plateaud, need to be made with caution. For MEP and MBP specifically, higher concentrations detected in urine of college-aged females in comparison to the general female population in surveys from 2005 to 2008 are no longer evident in later years (Fig 2A and 2C). DEP exposure is predominately through use of cosmetics and personal care products and potential indoor air sources [2]. Greater decline in college-aged female exposure in comparison to the general female population in NHANES likely reflects the differences in personal care products used between these groups, an inference also made for the general U.S. population given differential rates of MEP decline in adults and children from 2001–2010 [12]. Phthalate metabolites trend differently in time, however. Notably, MEP and MBP are lower in years after 2009 (One-way ANOVA with Tukey’s significance test, p<0.05) but MiBP has remained rather constant since 2005 (Fig 2A–2C). There are a variety of sources of DBP and DiBP; in past studies, DiBP exposure has been linked to diet and indoor/outdoor air and DBP exposure to diet and personal care product use [1, 37], while a fasting study discounted the contribution of diet for DBP and DiBP and suggested dust contributes to on-going exposures [38]. Declining trends in urinary biomarkers of phthalate exposure are likely due to the phasing out of phthalates, DEP and DBP in particular, in consumer products in response to advocacy efforts to raise consumer awareness and create a market for “phthalate-free” formulations, especially in cosmetics and personal care products [24, 39]. On the other hand, shifts in manufacturing may have increased use of DiBP as a replacement for DBP in consumer products in recent years, leading to the increase and fluctuation in MiBP concentrations observed in the U.S. population [24, 40].

**Fig 3:**
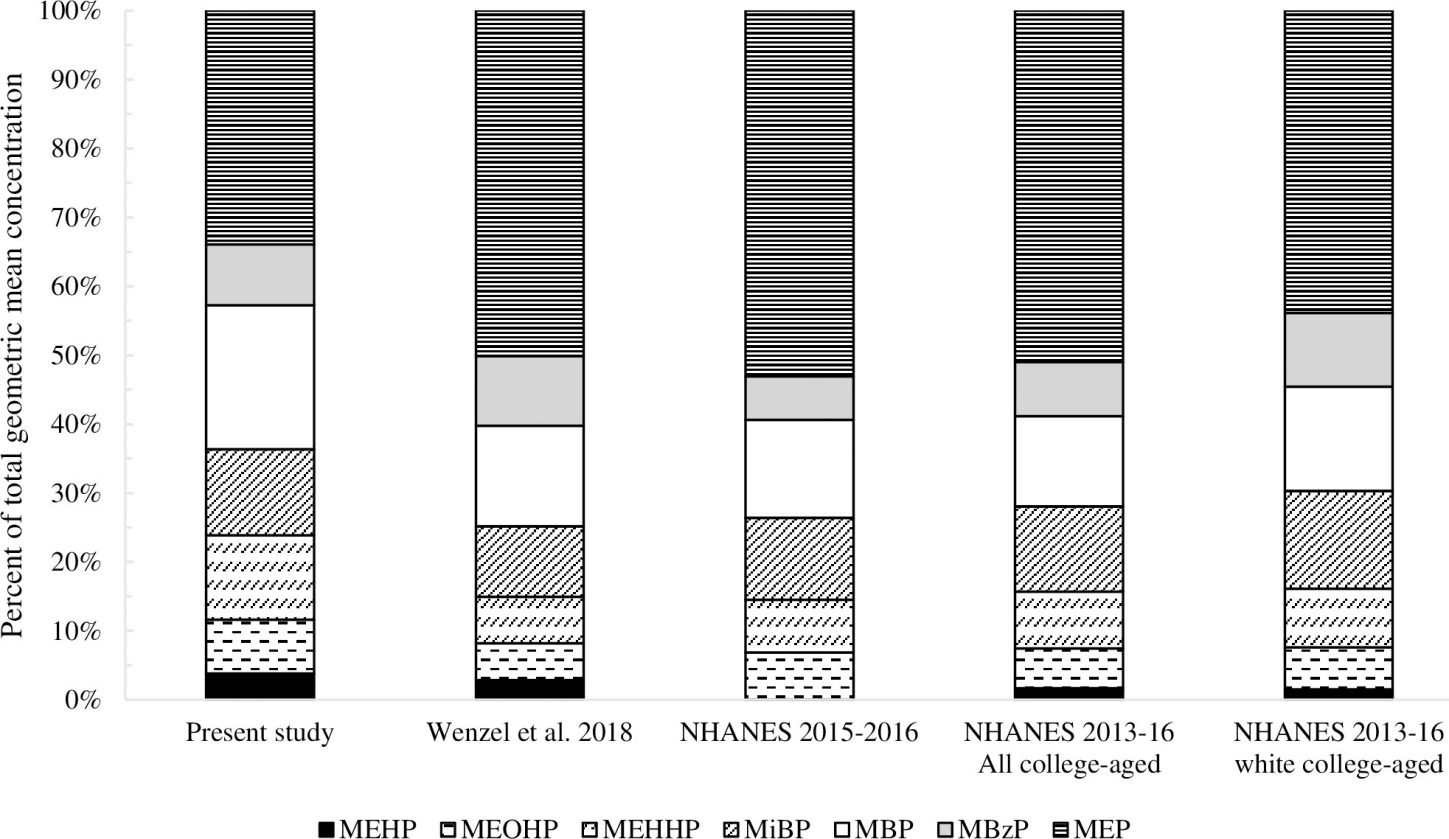
Distribution profile of phthalate metabolites using geometric mean concentrations. Female college students (present study, N = 215) are compared to pregnant adults in another local study by Wenzel et al. [17] (N = 378) and females sampled in NHANES in 2015–2016 (all age 3–80 years, N = 1501) as well as NHANES combined 2013–2014 and 2015–2016 cycles for All college-aged (18–22 years) (N = 184) and non-hispanic white college-aged (N = 56) females.

The distribution pattern of phthalate metabolites on average in the present study is compared to the reference populations of pregnant females from Charleston, SC in Wenzel et al. [17] and female groups sampled in recent years by NHANES in Fig 3. Survey data is presented as all females from NHANES 2015–2016 (age 3–80 years, N = 1501) and also as NHANES 2013–2014 and 2015–2016 for all College-aged females (N = 184) and non-hispanic white college-aged females (N = 56), since these closely compare to the demographic sampled in the present study. NHANES shares the racial and ethnic identity of participants in five options: Mexican American, other hispanic, non-hispanic white, non-hispanic Black and other race, including multi-racial. Two NHANES cycles were combined to assess college-aged females to increase sample size. Distribution patterns across these studies are similar, although the present study has a lower relative concentration of MEP. The distribution pattern of phthalate metabolites was most similar to the profile of the NHANES non-hispanic white college-aged female subset. Non-hispanic white college-aged women (N = 56) are observed to have lower MEP concentrations (p = 0.023) than non-hispanic Black college-age women (N = 48) in this NHANES sample, not controlling for other potential variables (One-way ANOVA with pairwise comparisons by Tukey’s test; all other phthalate metabolite levels are similar between groups). This observation supports previous studies indicating disparities in phthalate exposure by race and ethnicity, particularly higher exposure in African Americans to DEP [17].

#### Effect of sample timing on phthalate metabolite biomonitoring results

Sample collection timing, an important feature of biomonitoring study design, may influence cross-study comparisons for certain phthalate metabolites [12]. Particularly, we suspect that MEP and MiBP could be affected, considering that these are low-molecular weight phthalates and concentrations were lower than NHANES results. Whether time of sampling affects results depends on temporal exposure patterns and biotransformation and excretion rates. We did not know what to expect regarding college female exposure, such as through their use of personal care products, prior to this study due to a lack of information [25], and chose the late-afternoon/early evening (4–6 pm) for sample collection to accommodate the class and work schedule of students and clinicians. Timing of spot urine sampling in NHANES is provided in the database as taking place during morning, afternoon or evening sessions. Only 18% of females (2015–2016) and 19% of college-age females (NHANES 2013–2016) attended evening survey sessions, while most attended in the mornings (46% and 44%, respectively). Analyzed by sampling session, college-aged females (NHANES 2013–2016) had geometric mean concentrations of MEP and MiBP that were higher in the morning > afternoon > evening (S1 File; afternoon and evening were statistically significantly lower than morning for MiBP, One-way ANOVA with Tukey’s test, p<0.05). Interestingly, geometric mean concentrations were lower in the afternoon than the morning for the other phthalate metabolites measured as well (S1 File).

DEP is known to be a relatively rapidly excreted phthalate with exposure predominately through dermal or inhalation uptake from personal care product use. For instance, elimination half-life of MEP (2.1 h after inhalation and dermal exposure) has been reported to be 2–4 times faster than MEHP [11, 41]. Urinary elimination half-life has been reported for a single orally dosed adult male to be 2.6 h for MBP and 3.9 h for MiBP [42]. Intra-individual variation has been observed between multiple spot urine samples or between spot, early-morning voids and 24-hour samples for several phthalate metabolites since urine concentration is dependent on time elapsed after exposure, among other factors (e.g. number of urine voids and metabolic rate) [11, 13, 43, among others reviewed in 44]. Notably, Preau et al. observed MEP geometric mean concentrations among eight adults to be significantly lower in the evening (52.8 ng/mL, 6 pm-midnight) compared to the morning and afternoon (73 and 72 ng/mL, defined as midnight-noon and noon-6 pm, respectively) (p<0.01; factor difference of 0.73) [11]. About 20% of MEP variation observed in the study was attributed to intra-individual variability which was due to fluctuation within the day rather than between days, and cyclic patterns in MEP observed were attributed to routine personal care product use behaviors especially during the work week. Silva et al. also report MEP concentration in NHANES participants (1999–2000) to be lower in the evening (by ~0.7–0.8 times) compared to morning and midday after standardizing for differences in covariates across groups [23]. We also found that college-aged NHANES females (2013–2016) had geometric mean MEP concentrations that were 0.7–0.8 times lower in the evening than the morning and afternoon sessions (S1 File). Although not all phthalate metabolites reported in the present study were analyzed, it is significant that while MEP was lower, the other three phthalate metabolites studied (MBP, MEHP and MBzP) were higher in the evenings [23]. MiBP was not tracked in these studies [11, 23], so the record is incomplete regarding the effect of time of day for this phthalate metabolite, and this is further complicated by the multiple potential sources of DiBP exposure [24, 40]. We found that MiBP geometric mean concentrations were ~0.6–0.7 times lower in the evening than the morning and afternoon sessions for college-aged NHANES females (2013–2016) (not controlling for demographic variables, S1 File).

Since most urine samples were collected during the evenings, our study may report lower metabolite concentrations if exposure to DEP was predominately in the morning or the night before. In order not to miss peak excretion, it may be recommended that 24-h pooled samples be conducted rather than spot samples, however this can be burdensome for study subjects [31], depends on the objective of the exposure assessment and the phthalate metabolite considered [43] and was not feasible given the large sample size targeted in the present study. To compensate, we surveyed study participants on personal care product use behaviors with attention to timing (e.g. use within 6 hrs or 24 hrs) [25]. Braun et al. reported the importance of personal care product use timing and related the total product use within the last 6 hrs to higher urinary phthalate metabolites, especially for MEP and MBP [8]. Out of all personal care products surveyed for use in the present study (N = 35), on average students used 12 (IQR 8–15) within 24 hrs and 5 (IQR 2–8) within 6 hrs. Of five products that have been associated with MEP (deoderant, perfume, hand lotion, body lotion and shaving cream, after [8]), students with matched behavioral survey and urine data (N = 133) used 1.2 (IQR 0–2) within 6 hrs and 2.5 (IQR 1–3) within 24 hrs. Therefore, at least half of personal care product use for study participants was separated from the time of sampling by over 6 hours. Although we do not know the amounts of products used or their composition, and physiological variables that affect excretion rate are not modeled here due to data gaps, this time separation between product use and spot urine sample collection likely contributes significantly to the lower MEP levels observed. Over 80% of participants completing the behavioral survey were not aware whether their personal care products contained phthalates [25] (Fig 1).

## Conclusions

In summary, although relatively high phthalate metabolite concentrations were originally hypothesized for this college-aged female cohort given previously-reported high personal care product use [25], levels measured in urine samples collected in winter 2016–2017 were generally similar to the U.S. female population surveyed contemporaneously in 2015–2016 [22]. MEP concentrations in the present study, which were lower by about a factor of 2, may be explained by both demographic and sample collection timing factors. The timing of exposure, such as reported through personal care product use, relative to sample collection for spot samples is shown to influence results for this rapidly excreted chemical. Future studies may change sample collection to earlier in the day or collect multiple spot samples to test temporal reliability of phthalate urinalysis in young adults, particularly college students. Reconstructing daily intake estimates from biomonitoring data and identifying important exposure routes are also areas for future research. Demographic factors become important in cross-study comparisons due to the ways that preferences/behaviors/systems related to sociocultural and socioeconomic factors and metabolic transformation rates vary within the population and affect both exposure and urinalysis. Levels of phthalate-replacement chemicals, inclusion of a more diverse population in a focused college-aged biomonitoring study and systemic interventions to rectify exposure disparities are extensions of this work that are warranted given the vulnerability of this young adult group to potential reproductive and endocrine-disrupting chemicals.

## Supporting information

S1 Table Phthalate metabolite method detection limits. (TIF)

S2 Table Phthalate metabolite analysis QA/QC performance. (TIF)

S3 Table Sample size of NHANES data (all females) and college-aged female subset over time (data presented in Figs 2 and 3 and S1). (TIF)

S4 Table Reported educational level and race/ethnicity for the NHANES college-aged female subset (18–22 years old) in the 2013–2014 and 2015–2016 cycles, by number of individuals. (TIF)

S1 File NHANES college-aged female phthalate metabolite analysis by sampling time. Sample number in timing sessions for NHANES college-aged female subset (18–22 years old) by ethnicity in the combined 2013–2014 and 2015–2016 cycles. Geometric mean concentrations (ng/mL) with 95% confidence intervals (CI) of urinary phthalate metabolites for NHANES college-aged female subset (18–22 years old) (from Table 2) by sampling time of day (morning, afternoon or evening session).
(DOCX)

S2 File Tabulated individual urinalysis data in the present study. Unadjusted phthalate metabolite concentrations and specific gravity (S.G.) of urine collected from college women (2016–2017, N = 215) in the current study. Σ7 is the sum of MBP, MBzP, MEHHP, MEHP, MEOHP, MEP and MiBP. One outlier value of MBP was excluded from analysis (red text). Values highlighted in yellow were concentrations quantified below the limit of detection (LOD). For all phthalate metabolites except MNP, values are represented as LOD/sqrt2.
(DOCX)

S1 Fig Total phthalate metabolite concentrations measured in college-aged female NHANES participants (2005–2016). Geometric mean U.S. civilian demographic-weighted concentrations with 95% confidence intervals of the sum of 7 phthalate metabolites (MEP, MiBP, MBP, MEHP, MEOHP, MEHHP and MBzP) detected in urine sampled from college-aged females in the National Health and Nutrition Examination Survey (NHANES) cycles over time. Note, not all data are for women with college educations. Letters indicate significantly different years using log-transformed adjusted concentrations by ANOVA with Tukey’s significance test (p<0.05).
(TIF)
