# Supplementary figures and images for: Phthalate exposure among U.S. college-aged women: Biomonitoring in an undergraduate student cohort (2016-2017) and trends from the National Health and Examination Survey (NHANES, 2005-2016)
Source: PLoS One. 2022 Feb 11;17(2):e0263578. doi: 10.1371/journal.pone.0263578 (PMC8836309; doi:10.1371/journal.pone.0263578)

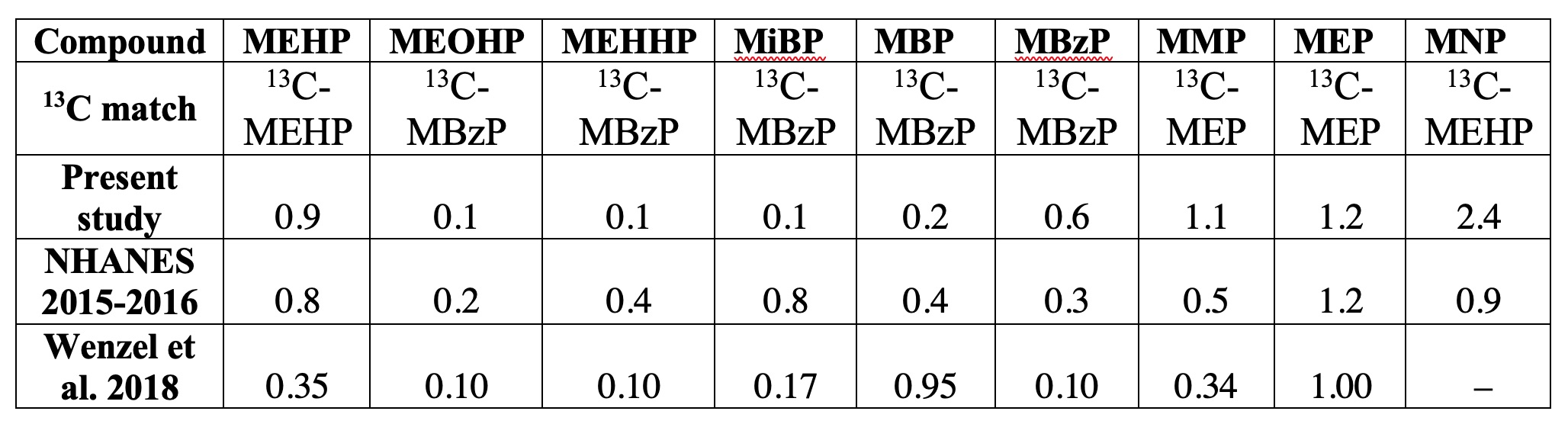

Supplement: S1 Table — (TIF) [file pone.0263578.s001.tif]

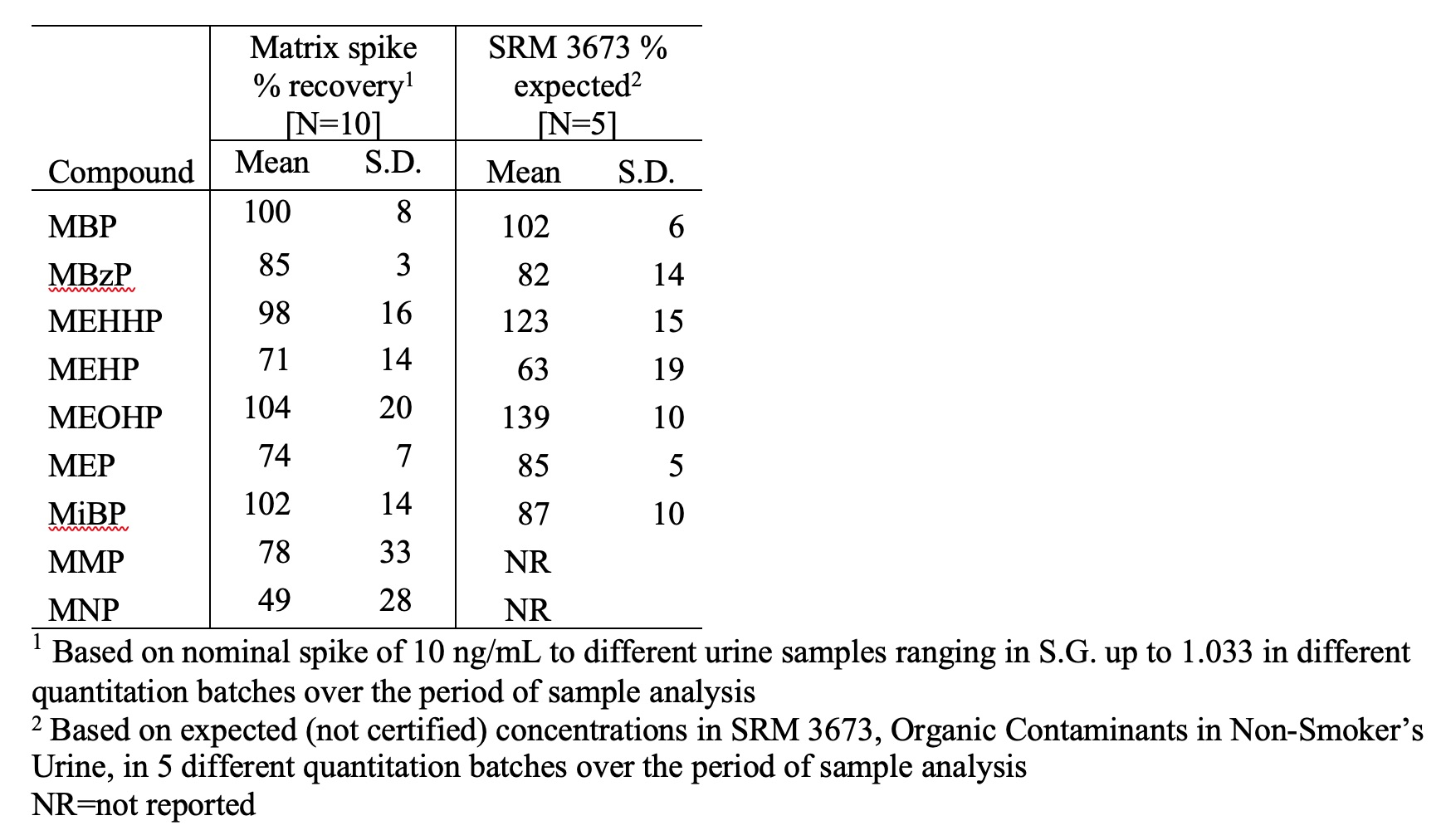

Supplement: S2 Table — (TIF) [file pone.0263578.s002.tif]

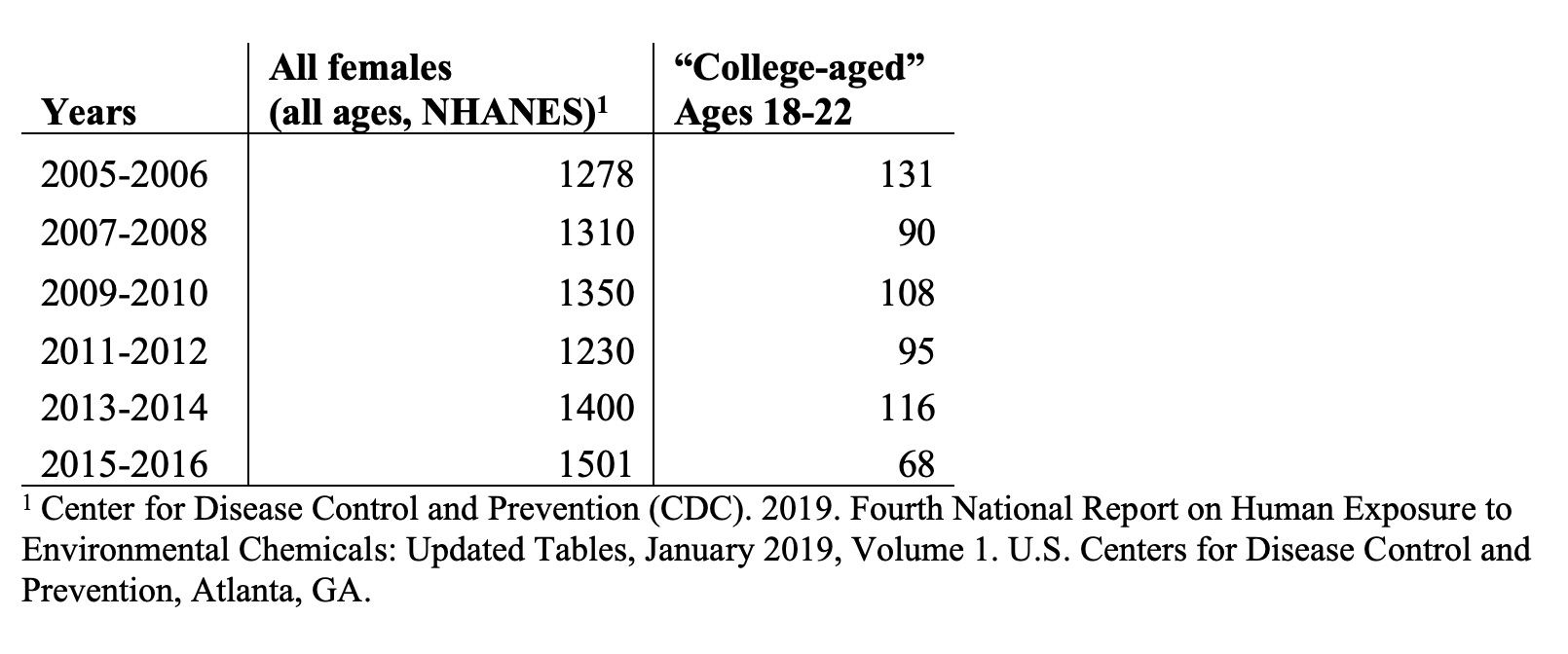

Supplement: S3 Table — (TIF) [file pone.0263578.s003.tif]

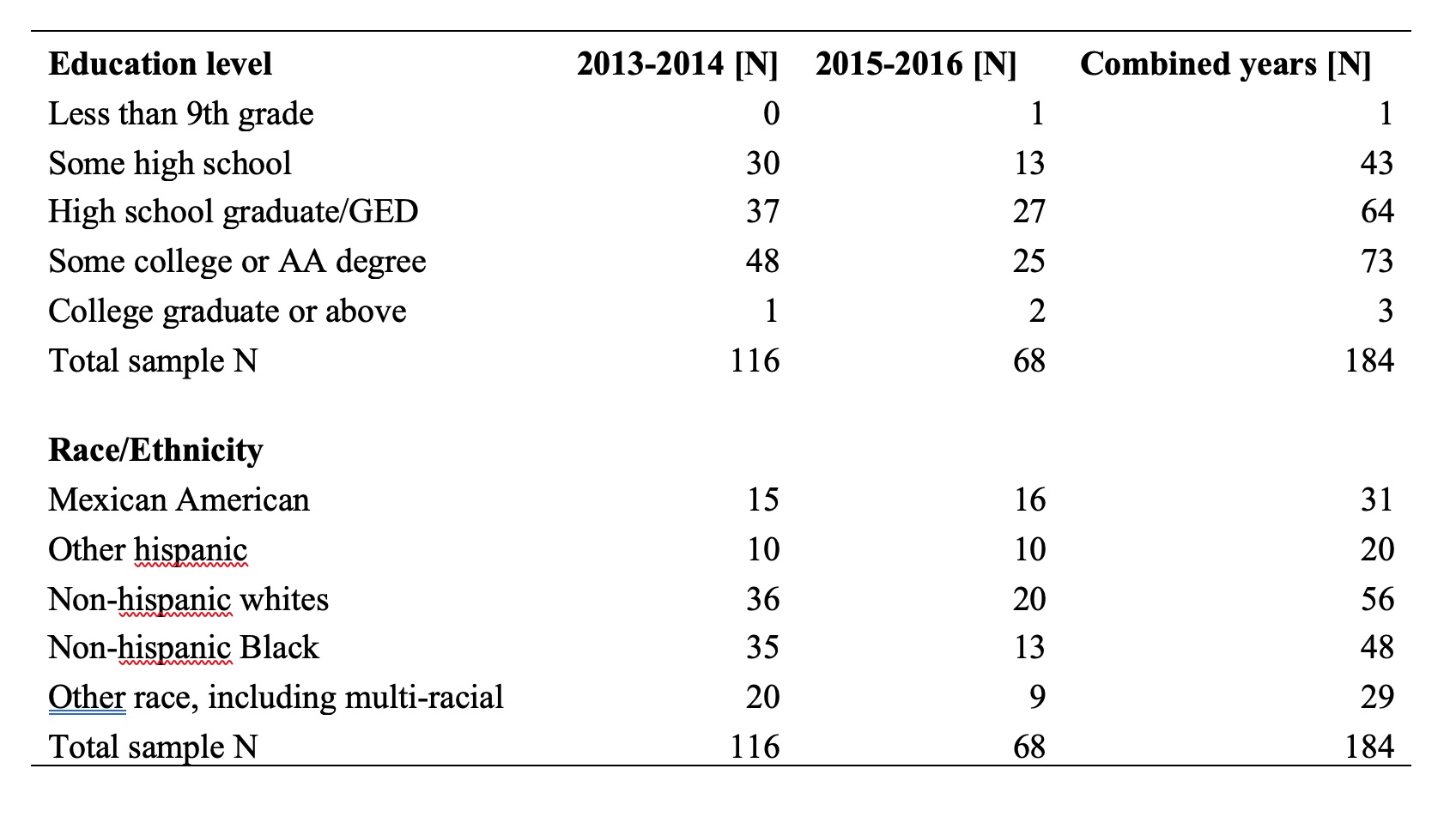

Supplement: S4 Table — (TIF) [file pone.0263578.s004.tif]

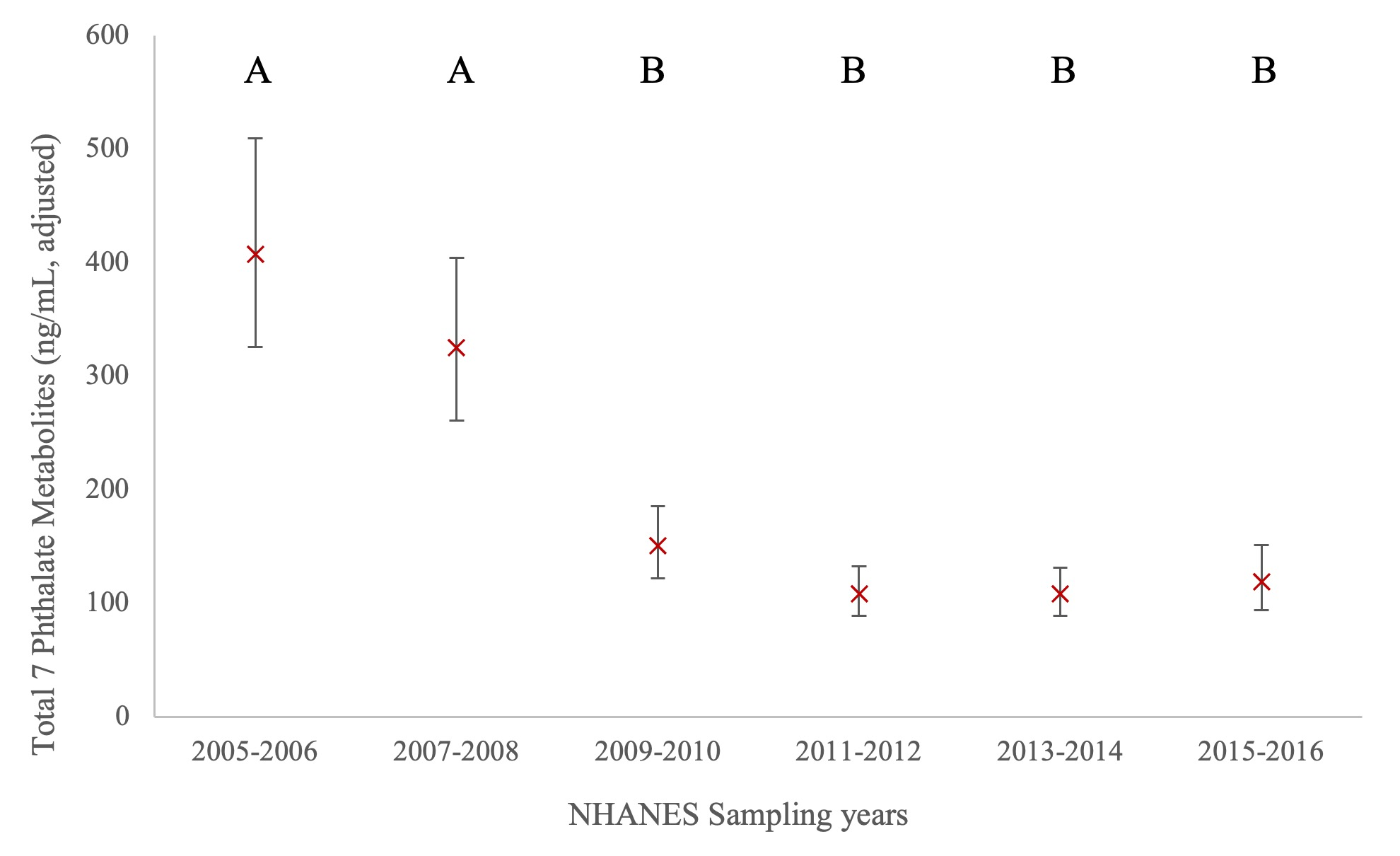

Supplement: S1 Fig — Geometric mean U.S. civilian demographic-weighted concentrations with 95% confidence intervals of the sum of 7 phthalate metabolites (MEP, MiBP, MBP, MEHP, MEOHP, MEHHP and MBzP) detected in urine sampled from college-aged females in the National Health and Nutrition Examination Survey (NHANES) cycles over time. Note, not all data are for women with college educations. Letters indicate significantly different years using log-transformed adjusted concentrations by ANOVA with Tukey’s significance test (p<0.05). (TIF) [file pone.0263578.s007.tif]
