# Supplementary material for: Phthalate exposure among U.S. college-aged women: Biomonitoring in an undergraduate student cohort (2016-2017) and trends from the National Health and Examination Survey (NHANES, 2005-2016)
Source: PLoS One. 2022 Feb 11;17(2):e0263578. doi: 10.1371/journal.pone.0263578 (PMC8836309; doi:10.1371/journal.pone.0263578)
Supplement: S1 File — Sample number in timing sessions for NHANES college-aged female subset (18–22 years old) by ethnicity in the combined 2013–2014 and 2015–2016 cycles. Geometric mean concentrations (ng/mL) with 95% confidence intervals (CI) of urinary phthalate metabolites for NHANES college-aged female subset (18–22 years old) (from Table 2) by sampling time of day (morning, afternoon or evening session). (DOCX) [file pone.0263578.s005.docx]

**S1 File. NHANES college-aged female phthalate metabolite analysis by sampling time**

Sample number timing session for NHANES college-aged female subset (18-22 years old) by ethnicity
in the combined 2013-2014 and 2015-2016 cycles

| **Race/Ethnicity** | **Sample timing [N]** | | |  |
| --- | --- | --- | --- | --- |
|  | **Morning** | **Afternoon** | **Evening** | **All** |
| Mexican American | 10 | 13 | 8 | 31 |
| Other hispanic | 9 | 7 | 4 | 20 |
| Non-hispanic whites | 28 | 19 | 9 | 56 |
| Non-hispanic Black | 24 | 17 | 7 | 48 |
| Other race, including multi-racial | 10 | 13 | 6 | 29 |
| Total sample N | 81 | 69 | 34 | 184 |

Geometric mean concentrations (ng/mL) with 95% confidence intervals (CI) of urinary phthalate metabolites for NHANES college-aged female subset (18-22 years old) (from Table 2) by sampling time of day (morning, afternoon or evening session)

| **Compound** | **NHANES 2013-16 college-aged females [N=184]** | | | **(95% CI)** | | **Morning [N=81]** | | **(95% CI)** | | **Afternoon [N=69]** | | | **(95% CI)** | | **Evening [N=34]** | | **(95% CI)** |
| --- | --- | --- | --- | --- | --- | --- | --- | --- | --- | --- | --- | --- | --- | --- | --- | --- | --- |
| MBP | 11.6 | (10.1-13.3) | | | | 15.5 | (12.4-19.2) | | | 9.54* | | (7.54-12.1) | | | 9.63^ | (6.89-13.5) | |
| MBzP | 6.99 | (5.92-8.26) | | | | 8.27 | (6.32-10.8) | | | 5.03* | | (3.76-6.74) | | | 6.05 | (3.99-9.16) | |
| MEHHP | 7.30 | (6.14-8.67) | | | | 9.78 | (7.49-12.8) | | | 5.66* | | (4.24-7.56) | | | 7.90 | (5.23-11.9) | |
| MEHP | x |  | | | |  |  | | |  | |  | | |  |  | |
| MEOHP | 5.11 | (4.32-6.04) | | | | 6.91 | (5.37-8.90) | | | 3.86* | | (2.94-5.08) | | | 5.77 | (3.91-8.52) | |
| MEP | 45.1 | (37.2-54.7) | | | | 55.7 | (40.7-76.2) | | | 49.5 | | (35.2-69.5) | | | 40.0 | (24.7-64.9) | |
| MiBP | 10.9 | (9.14-12.7) | | | | 14.4 | (11.5-17.9) | | | 9.34* | | (7.36-11.9) | | | 8.17* | (5.81-11.5) | |
| MMP | x |  | | | |  |  | | |  | |  | | |  |  | |
|  |  | |  | |  | | | |  | |  | | |  |  | |  |

* Statistically significantly different (ANOVA, Tukey’s HSD, p<0.05) compared to concentrations in morning samples; ^ p=0.054
x: Not reported by CDC (2019) because >40% of samples <LOD.
