# Supplementary material for: Phthalate exposure among U.S. college-aged women: Biomonitoring in an undergraduate student cohort (2016-2017) and trends from the National Health and Examination Survey (NHANES, 2005-2016)
Source: PLoS One. 2022 Feb 11;17(2):e0263578. doi: 10.1371/journal.pone.0263578 (PMC8836309; doi:10.1371/journal.pone.0263578)
Supplement: S2 File — Unadjusted phthalate metabolite concentrations and specific gravity (S.G.) of urine collected from college women (2016–2017, N = 215) in the current study. Σ7 is the sum of MBP, MBzP, MEHHP, MEHP, MEOHP, MEP and MiBP. One outlier value of MBP was excluded from analysis (red text). Values highlighted in yellow were concentrations quantified below the limit of detection (LOD). For all phthalate metabolites except MNP, values are represented as LOD/sqrt2. (DOCX) [file pone.0263578.s006.docx]

**S2 File. Tabulated individual urinalysis data in the present study.** Unadjusted phthalate metabolite concentrations and specific gravity (S.G.) of urine collected from college women (2016-2017, N=215) in the current study. $\Sigma$7 is the sum of MBP, MBzP, MEHHP, MEHP, MEOHP, MEP and MiBP. One outlier value of MBP was excluded from analysis (red text). Values highlighted in yellow were concentrations quantified below the limit of detection (LOD). For all phthalate metabolites except MNP, values are represented as LOD/sqrt2 (see Table S1 for limits of detection).

| **S.G.** | **Phthalate metabolite concentration, unadjusted for specific gravity (ng/mL)** | | | | | | | | | |
| --- | --- | --- | --- | --- | --- | --- | --- | --- | --- | --- |
|  | **MBP** | **MBzP** | **MEHHP** | **MEHP** | **MEOHP** | **MEP** | **MiBP** | **MMP** | **MNP** | $\boldsymbol{\Sigma}$**7** |
| 1.018 | 12.00 | 4.94 | 6.37 | 1.52 | 4.16 | 4.82 | 8.09 | 0.0 | 0.0 | 41.90 |
| 1.006 | 1.86 | 0.42 | 0.58 | 0.0 | 0.51 | 4.13 | 0.34 | 4.02 | 0.0 | 7.85 |
| 1.035 | 11299.85 | 22.90 | 7.56 | 2.19 | 5.55 | 23.90 | 8.30 | 0.0 | 0.0 | 70.40 |
| 1.034 | 16.45 | 3.55 | 9.64 | 1.06 | 5.82 | 19.10 | 19.60 | 0.78 | 0.0 | 75.22 |
| 1.049 | 34.55 | 23.70 | 22.90 | 5.52 | 13.50 | 38.90 | 14.90 | 1.58 | 0.0 | 153.97 |
| 1.002 | 0.0 | 1.04 | 1.86 | 0.0 | 0.57 | 0.85 | 0.0 | 0.0 | 0.0 | 4.32 |
| 1.008 | 3.77 | 3.87 | 2.47 | 0.0 | 1.62 | 4.82 | 4.58 | 0.0 | 0.0 | 21.13 |
| 1.024 | 20.95 | 10.30 | 7.78 | 0.64 | 3.72 | 296.00 | 10.90 | 0.78 | 0.0 | 350.29 |
| 1.028 | 23.10 | 12.90 | 8.97 | 2.25 | 8.45 | 17.22 | 7.43 | 0.0 | 0.0 | 80.32 |
| 1.037 | 10.10 | 2.39 | 9.71 | 2.44 | 6.43 | 7.97 | 6.29 | 0.0 | 0.0 | 45.33 |
| 1.035 | 12.80 | 1.44 | 11.30 | 2.72 | 6.32 | 42.33 | 16.20 | 0.0 | 0.0 | 93.11 |
| 1.019 | 6.36 | 2.66 | 4.26 | 0.0 | 2.55 | 18.70 | 6.22 | 0.78 | 0.0 | 40.75 |
| 1.024 | 10.60 | 4.31 | 2.63 | 0.0 | 1.58 | 2.23 | 2.77 | 0.0 | 0.0 | 24.12 |
| 1.005 | 2.55 | 0.42 | 2.19 | 0.0 | 1.44 | 6.96 | 1.67 | 0.0 | 0.0 | 15.23 |
| 1.024 | 15.10 | 6.21 | 6.12 | 0.0 | 3.73 | 10.03 | 8.95 | 0.0 | 0.0 | 50.14 |
| 1.024 | 6.99 | 1.66 | 6.54 | 0.0 | 4.50 | 5.36 | 8.28 | 0.78 | 0.0 | 33.33 |
| 1.022 | 8.35 | 10.10 | 10.70 | 1.02 | 5.46 | 6.24 | 11.30 | 0.0 | 0.0 | 53.17 |
| 1.019 | 2.36 | 0.42 | 4.55 | 0.0 | 2.62 | 2.88 | 1.17 | 0.78 | 0.0 | 14.00 |
| 1.018 | 10.40 | 5.25 | 8.01 | 0.64 | 4.64 | 9.88 | 7.19 | 0.78 | 0.0 | 46.01 |
| 1.038 | 9.80 | 5.29 | 8.00 | 0.0 | 5.51 | 6.60 | 4.08 | 0.78 | 0.0 | 39.28 |
| 1.048 | 24.80 | 10.40 | 27.90 | 13.50 | 22.00 | 13.03 | 33.50 | 0.78 | 0.0 | 145.13 |
| 1.026 | 14.15 | 21.10 | 11.20 | 1.78 | 7.23 | 27.70 | 6.00 | 2.65 | 0.0 | 89.16 |
| 1.013 | 2.79 | 0.88 | 1.20 | 0.0 | 0.89 | 6.61 | 1.53 | 0.0 | 0.0 | 13.90 |
| 1.007 | 1.72 | 0.42 | 2.01 | 0.0 | 1.24 | 0.85 | 1.65 | 0.78 | 0.0 | 7.89 |
| 1.031 | 12.80 | 6.99 | 20.50 | 1.73 | 16.60 | 408.52 | 9.10 | 0.0 | 0.0 | 476.24 |
| 1.042 | 71.80 | 230.00 | 11.60 | 1.47 | 7.99 | 35.50 | 39.40 | 5.77 | 0.0 | 397.76 |
| 1.008 | 5.65 | 0.42 | 4.16 | 0.0 | 2.39 | 3.96 | 3.02 | 0.78 | 0.0 | 19.60 |
| 1.007 | 3.80 | 1.06 | 0.83 | 0.0 | 0.44 | 3.96 | 4.52 | 2.97 | 0.0 | 14.61 |
| 1.015 | 5.42 | 1.98 | 8.33 | 0.64 | 4.64 | 4.87 | 6.16 | 1.72 | 0.0 | 32.04 |
| 1.026 | 7.77 | 17.60 | 14.30 | 0.64 | 9.06 | 52.80 | 3.62 | 0.78 | 0.0 | 105.79 |
| 1.025 | 16.35 | 13.90 | 7.80 | 0.64 | 5.95 | 1290.00 | 6.55 | 0.78 | 0.0 | 1341.19 |
| 1.037 | 9.59 | 5.65 | 4.90 | 0.64 | 2.77 | 18.90 | 3.99 | 0.78 | 0.0 | 46.44 |
| 1.007 | 2.77 | 1.48 | 1.66 | 0.0 | 1.09 | 20.82 | 1.89 | 0.0 | 0.0 | 29.71 |
| 1.007 | 1.42 | 0.98 | 0.65 | 0.0 | 0.42 | 3.23 | 0.47 | 0.0 | 0.0 | 7.17 |
| 1.021 | 13.90 | 4.93 | 6.68 | 0.0 | 3.28 | 10.70 | 9.53 | 2.98 | 0.0 | 49.02 |
| 1.03 | 12.00 | 5.99 | 4.79 | 0.0 | 4.20 | 42.30 | 7.34 | 0.78 | 0.0 | 76.62 |
| 1.035 | 14.40 | 15.50 | 49.20 | 7.19 | 33.70 | 7.75 | 5.76 | 0.78 | 0.11 | 133.50 |
| 1.031 | 34.75 | 4.87 | 15.90 | 0.88 | 7.86 | 10.30 | 10.20 | 5.02 | 0.0 | 84.76 |
| 1.045 | 37.55 | 10.70 | 32.70 | 9.26 | 20.20 | 120.00 | 30.90 | 5.23 | 0.0 | 261.31 |
| 1.01 | 33.80 | 4.28 | 4.36 | 0.0 | 2.99 | 4.69 | 8.76 | 0.78 | 0.0 | 58.88 |
| 1.024 | 13.20 | 2.50 | 6.80 | 3.46 | 4.63 | 20.72 | 21.70 | 4.73 | 0.0 | 73.01 |
| 1.013 | 2.31 | 3.12 | 1.81 | 0.0 | 1.43 | 15.42 | 0.98 | 0.0 | 0.0 | 25.07 |
| 1.006 | 1.35 | 0.42 | 1.18 | 0.0 | 0.61 | 1.51 | 1.31 | 5.59 | 0.0 | 6.38 |
| 1.041 | 42.00 | 10.80 | 14.70 | 2.14 | 14.00 | 32.53 | 23.50 | 0.0 | 0.0 | 139.67 |
| 1.036 | 33.40 | 19.20 | 7.55 | 2.90 | 5.79 | 147.52 | 10.30 | 0.0 | 0.0 | 226.66 |
| 1.009 | 2.30 | 0.0 | 0.91 | 0.0 | 0.58 | 1.53 | 0.30 | 0.0 | 0.0 | 5.62 |
| 1.032 | 34.20 | 20.00 | 12.30 | 1.71 | 9.49 | 42.82 | 17.50 | 4.53 | 0.0 | 138.02 |
| 1.006 | 4.28 | 1.15 | 0.77 | 0.0 | 0.49 | 1.24 | 1.51 | 0.0 | 0.0 | 9.44 |
| 1.038 | 17.60 | 10.30 | 4.15 | 0.0 | 3.04 | 62.43 | 6.24 | 0.78 | 0.0 | 103.76 |
| 1.041 | 45.00 | 6.70 | 5.80 | 6.83 | 4.65 | 16.23 | 15.70 | 0.0 | 0.0 | 100.91 |
| 1.045 | 15.20 | 5.79 | 25.70 | 2.62 | 17.00 | 44.42 | 12.70 | 0.0 | 0.0 | 123.43 |
| 1.016 | 25.30 | 10.40 | 2.03 | 0.0 | 1.09 | 25.33 | 8.27 | 0.0 | 0.0 | 72.42 |
| 1.01 | 6.04 | 0.42 | 2.07 | 0.0 | 1.47 | 172.00 | 3.04 | 3.05 | 0.0 | 185.04 |
| 1.027 | 40.40 | 29.70 | 20.20 | 7.94 | 10.20 | 8.38 | 18.40 | 0.0 | 0.0 | 135.22 |
| 1.005 | 1.19 | 1.28 | 1.19 | 0.0 | 0.64 | 0.85 | 0.69 | 0.78 | 0.0 | 5.83 |
| 1.039 | 32.80 | 25.50 | 7.74 | 0.93 | 4.54 | 40.63 | 14.40 | 5.04 | 0.0 | 126.55 |
| 1.019 | 18.90 | 8.10 | 5.20 | 0.0 | 3.17 | 22.20 | 5.11 | 0.78 | 0.0 | 62.68 |
| 1.006 | 3.40 | 1.37 | 6.97 | 0.0 | 5.24 | 91.20 | 3.34 | 0.78 | 0.0 | 111.52 |
| 1.012 | 2.66 | 1.02 | 1.67 | 0.0 | 1.47 | 16.52 | 0.98 | 0.0 | 0.0 | 24.33 |
| 1.029 | 67.90 | 4.62 | 7.48 | 3.06 | 3.89 | 20.03 | 35.90 | 0.78 | 0.0 | 142.88 |
| 1.041 | 28.20 | 8.63 | 7.15 | 0.64 | 6.10 | 37.70 | 9.07 | 1.36 | 0.0 | 97.49 |
| 1.045 | 35.35 | 13.60 | 12.40 | 2.20 | 7.42 | 11.30 | 11.60 | 0.78 | 0.0 | 93.87 |
| 1.004 | 0.43 | 1.06 | 0.72 | 0.0 | 0.68 | 0.85 | 0.56 | 0.0 | 0.0 | 4.29 |
| 1.029 | 42.80 | 21.30 | 11.30 | 1.17 | 6.71 | 94.32 | 13.70 | 0.0 | 0.0 | 191.30 |
| 1.026 | 6.03 | 0.70 | 6.74 | 0.0 | 4.03 | 4.68 | 6.32 | 0.78 | 0.0 | 28.50 |
| 1.04 | 37.50 | 3.32 | 56.30 | 3.23 | 33.10 | 151.52 | 39.30 | 0.0 | 1.55 | 324.27 |
| 1.018 | 9.80 | 5.55 | 3.06 | 0.64 | 1.87 | 15.10 | 3.54 | 0.0 | 0.0 | 39.56 |
| 1.019 | 4.90 | 3.13 | 2.05 | 1.63 | 1.34 | 8.21 | 3.09 | 0.0 | 0.0 | 24.35 |
| 1.034 | 37.40 | 6.68 | 24.80 | 1.26 | 18.60 | 58.12 | 8.91 | 0.0 | 0.0 | 155.77 |
| 1.031 | 5.66 | 3.06 | 3.65 | 0.64 | 1.90 | 120.52 | 4.32 | 0.0 | 0.0 | 139.74 |
| 1.039 | 70.30 | 12.30 | 21.00 | 4.75 | 13.40 | 120.00 | 33.10 | 0.78 | 0.0 | 274.85 |
| 1.024 | 9.41 | 6.21 | 4.71 | 2.62 | 2.40 | 66.50 | 4.76 | 0.78 | 0.0 | 96.61 |
| 1.013 | 5.91 | 10.50 | 4.01 | 0.0 | 2.13 | 126.00 | 3.62 | 0.78 | 0.0 | 152.17 |
| 1.009 | 5.28 | 2.51 | 2.32 | 0.0 | 1.39 | 3.70 | 2.85 | 0.78 | 0.0 | 18.05 |
| 1.017 | 12.90 | 2.92 | 15.20 | 0.86 | 11.10 | 6.11 | 12.10 | 0.0 | 0.0 | 61.18 |
| 1.008 | 2.89 | 1.21 | 3.86 | 0.0 | 2.48 | 25.00 | 0.87 | 0.0 | 0.0 | 36.31 |
| 1.028 | 16.40 | 16.80 | 7.71 | 1.12 | 4.50 | 87.70 | 4.98 | 0.0 | 0.0 | 139.21 |
| 1.042 | 32.20 | 10.20 | 26.00 | 7.09 | 18.20 | 17.00 | 34.90 | 0.0 | 0.0 | 145.59 |
| 1.014 | 4.87 | 4.14 | 2.14 | 0.0 | 1.81 | 23.70 | 2.05 | 0.78 | 0.0 | 38.71 |
| 1.012 | 1.90 | 0.42 | 2.53 | 0.0 | 1.55 | 1.98 | 1.55 | 0.0 | 0.0 | 9.93 |
| 1.026 | 11.60 | 4.69 | 22.40 | 3.53 | 16.20 | 35.72 | 8.85 | 0.0 | 0.0 | 102.99 |
| 1.05 | 19.20 | 22.20 | 3.89 | 1.80 | 3.53 | 29.92 | 16.40 | 0.78 | 0.0 | 96.94 |
| 1.045 | 39.50 | 2.90 | 30.80 | 4.64 | 19.70 | 44.60 | 40.10 | 0.78 | 0.0 | 182.24 |
| 1.011 | 4.51 | 3.02 | 1.54 | 0.0 | 1.03 | 2.01 | 2.81 | 0.78 | 0.0 | 14.92 |
| 1.012 | 6.59 | 1.87 | 4.44 | 0.0 | 3.38 | 5.15 | 6.36 | 0.0 | 0.0 | 27.79 |
| 1.028 | 3.41 | 2.55 | 2.56 | 0.64 | 1.78 | 11.00 | 4.27 | 0.78 | 0.0 | 26.21 |
| 1.037 | 11.20 | 6.05 | 5.20 | 1.29 | 3.51 | 27.70 | 18.30 | 0.78 | 0.0 | 73.25 |
| 1.03 | 31.20 | 12.50 | 15.30 | 7.29 | 4.23 | 60.00 | 11.00 | 0.0 | 0.0 | 141.52 |
| 1.007 | 6.10 | 0.42 | 0.45 | 0.0 | 0.39 | 13.70 | 0.78 | 0.78 | 0.0 | 21.84 |
| 1.03 | 22.50 | 5.14 | 19.10 | 3.37 | 12.60 | 39.50 | 10.00 | 0.0 | 0.0 | 112.21 |
| 1.02 | 5.18 | 2.93 | 3.52 | 0.0 | 2.32 | 8.27 | 2.81 | 0.0 | 0.0 | 25.03 |
| 1.013 | 4.48 | 2.74 | 6.68 | 0.0 | 4.99 | 47.00 | 4.98 | 0.0 | 0.0 | 70.87 |
| 1.028 | 35.10 | 20.20 | 7.65 | 1.59 | 6.35 | 98.00 | 28.00 | 2.49 | 0.0 | 196.89 |
| 1.01 | 3.92 | 4.04 | 3.87 | 0.0 | 2.14 | 27.72 | 1.62 | 0.0 | 0.0 | 43.31 |
| 1.004 | 1.22 | 1.00 | 2.66 | 0.0 | 1.23 | 3.56 | 1.23 | 0.0 | 0.0 | 10.89 |
| 1.028 | 20.20 | 6.16 | 25.70 | 2.75 | 16.20 | 32.90 | 8.21 | 2.55 | 0.0 | 112.12 |
| 1.047 | 22.80 | 18.80 | 4.97 | 1.41 | 3.26 | 17.83 | 15.40 | 0.0 | 0.0 | 84.47 |
| 1.009 | 2.89 | 2.57 | 1.73 | 0.0 | 0.75 | 1.70 | 1.27 | 0.78 | 0.0 | 10.91 |
| 1.023 | 7.41 | 2.95 | 5.62 | 0.0 | 4.04 | 40.10 | 7.08 | 0.78 | 0.0 | 67.20 |
| 1.048 | 12.20 | 5.39 | 6.14 | 0.0 | 3.76 | 58.10 | 3.93 | 1.85 | 0.0 | 89.52 |
| 1.036 | 20.30 | 15.60 | 13.60 | 2.14 | 7.96 | 10.43 | 17.90 | 2.98 | 0.0 | 87.93 |
| 1.027 | 24.60 | 5.03 | 14.60 | 1.09 | 12.40 | 19.22 | 18.50 | 0.0 | 0.0 | 95.44 |
| 1.009 | 1.44 | 2.81 | 1.75 | 0.0 | 0.98 | 11.40 | 0.68 | 0.78 | 0.0 | 19.05 |
| 1.033 | 32.05 | 15.10 | 38.00 | 4.26 | 28.70 | 22.30 | 13.60 | 56.70 | 0.0 | 154.01 |
| 1.021 | 8.84 | 5.25 | 3.50 | 0.0 | 1.99 | 16.73 | 5.47 | 0.0 | 0.0 | 41.78 |
| 1.029 | 6.12 | 2.97 | 3.71 | 0.64 | 3.04 | 22.82 | 2.99 | 2.03 | 0.0 | 42.28 |
| 1.017 | 15.50 | 23.10 | 4.41 | 0.64 | 2.74 | 21.22 | 6.38 | 0.0 | 0.0 | 73.98 |
| 1.03 | 15.60 | 6.31 | 9.23 | 0.0 | 7.27 | 21.60 | 7.70 | 3.09 | 0.0 | 67.71 |
| 1.046 | 36.30 | 14.90 | 13.30 | 1.94 | 8.48 | 28.60 | 22.30 | 0.78 | 0.0 | 125.82 |
| 1.028 | 51.30 | 22.70 | 7.12 | 0.0 | 4.87 | 13.83 | 20.70 | 0.0 | 0.0 | 120.52 |
| 1.014 | 7.13 | 2.95 | 4.52 | 0.0 | 2.18 | 9.54 | 6.21 | 0.0 | 0.0 | 32.53 |
| 1.027 | 14.00 | 7.15 | 4.05 | 0.0 | 3.05 | 25.90 | 5.12 | 2.77 | 0.0 | 59.27 |
| 1.03 | 12.90 | 8.37 | 11.90 | 1.44 | 6.85 | 16.20 | 17.80 | 0.78 | 0.0 | 75.46 |
| 1.033 | 5.39 | 0.76 | 6.40 | 1.60 | 2.45 | 6.40 | 3.41 | 0.0 | 0.0 | 26.41 |
| 1.025 | 6.71 | 3.24 | 5.14 | 0.64 | 3.56 | 4.56 | 1.65 | 0.78 | 0.0 | 25.50 |
| 1.036 | 21.20 | 23.30 | 11.10 | 0.0 | 7.01 | 20.50 | 15.90 | 6.58 | 0.0 | 99.02 |
| 1.014 | 4.97 | 2.46 | 7.21 | 0.0 | 4.64 | 2.90 | 1.91 | 0.78 | 0.0 | 24.09 |
| 1.032 | 7.93 | 2.05 | 5.90 | 0.0 | 3.28 | 9.67 | 21.10 | 0.78 | 0.0 | 49.93 |
| 1.025 | 5.58 | 3.26 | 4.59 | 0.64 | 3.65 | 5.38 | 3.73 | 0.0 | 0.0 | 26.82 |
| 1.027 | 13.40 | 7.37 | 15.40 | 2.93 | 7.61 | 15.02 | 9.11 | 0.0 | 0.0 | 70.84 |
| 1.012 | 1.20 | 1.14 | 3.10 | 0.0 | 2.72 | 7.22 | 0.78 | 0.0 | 0.0 | 16.16 |
| 1.039 | 8.79 | 1.26 | 7.49 | 4.09 | 5.30 | 16.60 | 8.22 | 0.0 | 0.0 | 51.75 |
| 1.016 | 7.95 | 5.04 | 5.71 | 0.0 | 3.36 | 3.17 | 2.48 | 0.0 | 0.0 | 27.71 |
| 1.026 | 12.00 | 26.90 | 6.96 | 1.30 | 4.57 | 57.12 | 10.20 | 0.0 | 0.0 | 119.05 |
| 1.004 | 1.12 | 1.06 | 1.76 | 0.0 | 1.09 | 5.85 | 3.31 | 0.0 | 0.0 | 14.19 |
| 1.044 | 22.70 | 9.84 | 3.36 | 0.0 | 2.74 | 7.73 | 6.09 | 0.0 | 0.0 | 52.46 |
| 1.04 | 46.30 | 28.80 | 11.10 | 0.64 | 11.30 | 120.13 | 38.40 | 1.12 | 0.0 | 256.67 |
| 1.029 | 19.80 | 3.91 | 13.80 | 1.86 | 10.40 | 27.12 | 14.60 | 0.0 | 0.0 | 91.49 |
| 1.006 | 1.28 | 0.42 | 0.79 | 0.0 | 0.40 | 2.63 | 0.94 | 0.0 | 0.0 | 6.45 |
| 1.046 | 17.20 | 8.13 | 6.90 | 2.10 | 4.27 | 28.82 | 13.80 | 0.78 | 0.0 | 81.22 |
| 1.019 | 16.20 | 5.86 | 4.30 | 0.0 | 2.97 | 37.80 | 2.60 | 0.0 | 0.0 | 69.73 |
| 1.006 | 0.91 | 0.0 | 1.18 | 0.0 | 0.65 | 29.10 | 0.46 | 3.91 | 0.0 | 32.30 |
| 1.021 | 5.97 | 0.76 | 15.60 | 0.0 | 11.50 | 7.54 | 4.10 | 0.0 | 0.0 | 45.47 |
| 1.02 | 4.61 | 2.72 | 4.75 | 1.34 | 2.40 | 8.01 | 21.20 | 0.0 | 0.0 | 45.03 |
| 1.018 | 21.30 | 1.14 | 10.40 | 0.0 | 6.15 | 5.72 | 7.23 | 0.78 | 0.0 | 51.94 |
| 1.029 | 6.88 | 6.24 | 6.04 | 0.64 | 2.86 | 3.38 | 23.90 | 0.0 | 0.0 | 49.94 |
| 1.042 | 41.00 | 11.50 | 30.80 | 6.98 | 16.90 | 114.52 | 80.60 | 0.0 | 0.0 | 302.30 |
| 1.033 | 24.40 | 21.20 | 13.30 | 1.87 | 10.30 | 19.20 | 11.80 | 5.35 | 0.0 | 102.07 |
| 1.014 | 12.45 | 9.80 | 7.39 | 1.19 | 4.30 | 4.26 | 6.49 | 0.0 | 0.0 | 45.88 |
| 1.013 | 8.57 | 0.92 | 2.03 | 0.0 | 0.93 | 2.05 | 0.87 | 0.0 | 0.0 | 15.37 |
| 1.036 | 16.60 | 4.08 | 16.50 | 5.74 | 10.20 | 13.50 | 7.53 | 8.10 | 0.0 | 74.15 |
| 1.013 | 2.58 | 0.42 | 2.85 | 0.0 | 1.50 | 4.20 | 1.52 | 0.78 | 0.0 | 13.07 |
| 1.029 | 7.50 | 3.17 | 8.38 | 2.01 | 6.45 | 103.52 | 6.77 | 0.0 | 0.0 | 137.80 |
| 1.033 | 8.39 | 4.70 | 7.82 | 2.34 | 3.90 | 203.13 | 11.90 | 2.12 | 0.0 | 242.18 |
| 1.04 | 40.00 | 15.90 | 14.00 | 2.12 | 8.98 | 12.13 | 15.90 | 19.10 | 0.0 | 109.03 |
| 1.035 | 9.50 | 6.69 | 10.40 | 1.96 | 8.98 | 394.52 | 4.82 | 0.0 | 0.0 | 436.87 |
| 1.04 | 20.40 | 5.90 | 21.00 | 1.99 | 14.60 | 12.62 | 18.30 | 0.0 | 0.0 | 94.81 |
| 1.037 | 22.80 | 4.47 | 8.89 | 0.88 | 7.64 | 4.02 | 16.60 | 0.0 | 0.0 | 65.31 |
| 1.046 | 22.70 | 7.68 | 20.20 | 6.29 | 13.70 | 82.50 | 14.80 | 4.34 | 0.0 | 167.87 |
| 1.014 | 4.90 | 10.10 | 1.84 | 0.0 | 1.32 | 7.25 | 1.70 | 1.65 | 0.0 | 27.11 |
| 1.027 | 10.50 | 13.40 | 4.62 | 0.0 | 3.09 | 32.20 | 4.09 | 0.0 | 0.0 | 67.90 |
| 1.036 | 10.10 | 4.08 | 9.88 | 1.12 | 4.85 | 13.20 | 4.53 | 0.78 | 0.0 | 47.76 |
| 1.008 | 1.20 | 0.42 | 0.79 | 0.0 | 0.48 | 26.90 | 3.63 | 0.78 | 0.0 | 33.43 |
| 1.029 | 15.45 | 3.06 | 3.86 | 0.64 | 3.40 | 79.20 | 23.60 | 0.0 | 0.0 | 129.21 |
| 1.032 | 7.74 | 7.92 | 7.68 | 0.0 | 4.05 | 8.50 | 21.10 | 0.0 | 0.0 | 56.99 |
| 1.02 | 12.60 | 6.14 | 15.60 | 1.00 | 8.43 | 102.52 | 6.10 | 0.0 | 0.0 | 152.38 |
| 1.03 | 8.06 | 19.50 | 12.80 | 0.0 | 7.08 | 17.50 | 3.05 | 1.13 | 0.0 | 67.99 |
| 1.028 | 17.30 | 6.56 | 3.98 | 0.0 | 1.97 | 15.12 | 2.42 | 0.0 | 0.0 | 47.35 |
| 1.037 | 25.85 | 2.63 | 10.70 | 0.64 | 5.98 | 4.19 | 14.90 | 0.0 | 0.0 | 64.89 |
| 1.037 | 13.20 | 6.48 | 5.93 | 0.64 | 3.88 | 32.10 | 9.85 | 3.12 | 0.0 | 72.08 |
| 1.029 | 13.40 | 9.62 | 5.49 | 0.0 | 3.87 | 32.50 | 7.78 | 0.78 | 0.0 | 72.66 |
| 1.04 | 36.50 | 8.16 | 49.40 | 5.96 | 42.70 | 64.63 | 28.60 | 0.0 | 0.0 | 235.95 |
| 1.006 | 3.54 | 0.42 | 1.35 | 0.0 | 0.92 | 24.40 | 2.40 | 1.18 | 0.0 | 33.04 |
| 1.003 | 1.63 | 0.42 | 0.54 | 0.0 | 0.39 | 0.85 | 0.62 | 0.78 | 0.0 | 4.44 |
| 1.03 | 4.54 | 0.68 | 2.95 | 0.0 | 1.36 | 6.77 | 2.83 | 0.0 | 0.0 | 19.14 |
| 1.035 | 7.89 | 6.66 | 5.93 | 0.0 | 3.46 | 149.00 | 8.57 | 0.78 | 0.0 | 181.51 |
| 1.04 | 33.00 | 9.23 | 4.64 | 0.96 | 3.34 | 85.93 | 17.80 | 0.0 | 0.0 | 154.90 |
| 1.01 | 5.34 | 4.10 | 4.15 | 0.0 | 2.25 | 2.97 | 1.56 | 0.78 | 0.0 | 20.37 |
| 1.031 | 10.60 | 3.67 | 5.15 | 1.58 | 2.48 | 4.58 | 5.61 | 0.0 | 0.0 | 33.67 |
| 1.052 | 25.15 | 17.60 | 13.30 | 2.26 | 6.86 | 41.70 | 127.00 | 1.17 | 0.0 | 233.87 |
| 1.03 | 13.70 | 3.48 | 7.41 | 0.64 | 5.54 | 36.10 | 9.80 | 0.0 | 0.0 | 76.67 |
| 1.008 | 8.46 | 1.74 | 1.84 | 0.0 | 1.16 | 8.00 | 2.00 | 0.78 | 0.0 | 23.20 |
| 1.043 | 19.00 | 2.65 | 7.07 | 4.06 | 5.42 | 25.90 | 12.60 | 2.15 | 0.0 | 76.70 |
| 1.028 | 49.30 | 2.40 | 8.00 | 0.64 | 6.33 | 13.02 | 12.10 | 0.0 | 0.0 | 91.78 |
| 1.03 | 14.70 | 3.55 | 5.75 | 0.0 | 4.22 | 14.40 | 8.76 | 0.78 | 0.0 | 51.38 |
| 1.023 | 10.60 | 7.15 | 4.92 | 1.13 | 2.66 | 20.30 | 7.67 | 0.0 | 0.0 | 54.43 |
| 1.039 | 7.38 | 5.74 | 5.10 | 0.0 | 3.51 | 53.10 | 2.84 | 0.78 | 0.0 | 77.67 |
| 1.015 | 4.27 | 3.56 | 3.67 | 0.0 | 2.69 | 4.51 | 3.06 | 0.78 | 0.0 | 21.76 |
| 1.005 | 1.82 | 1.26 | 1.95 | 0.0 | 1.15 | 41.92 | 2.36 | 0.0 | 0.0 | 50.46 |
| 1.015 | 7.96 | 2.81 | 4.96 | 0.0 | 2.97 | 5.62 | 4.58 | 2.59 | 0.0 | 28.90 |
| 1.013 | 1.18 | 1.03 | 2.82 | 0.0 | 1.46 | 2.49 | 1.05 | 0.0 | 0.0 | 10.03 |
| 1.013 | 8.05 | 3.14 | 2.36 | 0.0 | 1.20 | 11.73 | 9.57 | 0.0 | 0.0 | 36.05 |
| 1.036 | 24.30 | 14.40 | 14.30 | 1.72 | 10.60 | 13.60 | 4.95 | 0.78 | 0.0 | 83.87 |
| 1.045 | 40.00 | 41.00 | 26.40 | 9.25 | 20.50 | 14.40 | 10.10 | 0.0 | 0.0 | 161.65 |
| 1.025 | 28.00 | 6.12 | 16.10 | 4.51 | 10.70 | 41.70 | 14.60 | 1.40 | 0.0 | 121.73 |
| 1.024 | 20.00 | 5.18 | 14.70 | 1.66 | 8.38 | 148.00 | 10.50 | 1.56 | 0.0 | 208.42 |
| 1.014 | 3.96 | 0.82 | 2.19 | 0.0 | 0.98 | 5.26 | 1.39 | 0.78 | 0.0 | 14.60 |
| 1.026 | 7.84 | 11.50 | 2.87 | 0.0 | 1.91 | 3.29 | 5.95 | 2.35 | 0.0 | 33.36 |
| 1.011 | 5.98 | 6.11 | 4.20 | 0.0 | 2.06 | 6.43 | 9.35 | 12.80 | 0.0 | 34.13 |
| 1.029 | 6.97 | 1.94 | 10.60 | 1.05 | 5.83 | 187.00 | 3.27 | 0.78 | 0.0 | 216.66 |
| 1.004 | 0.0 | 0.42 | 1.83 | 0.0 | 0.92 | 11.00 | 0.60 | 0.78 | 0.0 | 14.78 |
| 1.028 | 83.20 | 8.06 | 22.20 | 1.80 | 14.50 | 94.83 | 24.40 | 0.78 | 0.0 | 248.99 |
| 1.019 | 7.45 | 4.74 | 2.59 | 0.64 | 1.39 | 2.11 | 2.15 | 0.78 | 0.0 | 21.07 |
| 1.02 | 11.80 | 3.45 | 7.05 | 0.64 | 4.08 | 174.52 | 7.72 | 0.0 | 0.0 | 209.25 |
| 1.022 | 4.07 | 0.89 | 1.94 | 0.0 | 1.04 | 6.91 | 6.63 | 0.78 | 0.0 | 21.48 |
| 1.009 | 5.03 | 3.80 | 2.68 | 0.0 | 1.64 | 3.18 | 2.60 | 0.78 | 0.0 | 18.93 |
| 1.024 | 8.36 | 3.34 | 4.99 | 0.0 | 3.17 | 20.90 | 3.32 | 0.78 | 0.0 | 44.08 |
| 1.041 | 14.00 | 3.08 | 200.00 | 44.60 | 134.00 | 54.80 | 26.90 | 12.90 | 1.39 | 477.38 |
| 1.016 | 8.38 | 8.09 | 6.65 | 0.0 | 3.30 | 11.30 | 5.42 | 1.23 | 0.0 | 43.14 |
| 1.03 | 10.30 | 11.40 | 16.80 | 0.0 | 10.10 | 1030.00 | 7.26 | 0.0 | 0.0 | 1085.86 |
| 1.024 | 11.25 | 11.40 | 7.50 | 0.64 | 4.60 | 23.00 | 5.68 | 4.60 | 0.0 | 64.07 |
| 1.021 | 5.68 | 4.09 | 3.58 | 0.0 | 2.14 | 1.48 | 2.88 | 0.0 | 0.0 | 19.85 |
| 1.01 | 3.09 | 1.83 | 2.66 | 0.0 | 2.02 | 1.89 | 1.82 | 0.78 | 0.0 | 13.31 |
| 1.025 | 8.37 | 1.78 | 6.80 | 2.00 | 4.27 | 10.10 | 5.64 | 0.78 | 0.0 | 38.96 |
| 1.025 | 13.90 | 16.20 | 7.70 | 0.0 | 4.58 | 38.90 | 6.78 | 0.78 | 0.0 | 88.06 |
| 1.004 | 5.21 | 0.93 | 1.99 | 0.0 | 1.30 | 3.36 | 3.03 | 0.0 | 0.0 | 15.81 |
| 1.034 | 11.10 | 4.98 | 9.25 | 1.47 | 7.51 | 46.62 | 8.36 | 0.78 | 0.0 | 89.29 |
| 1.042 | 18.70 | 9.79 | 19.30 | 1.28 | 14.20 | 31.50 | 46.30 | 0.0 | 0.0 | 141.07 |
| 1.008 | 4.27 | 0.42 | 1.73 | 0.0 | 1.38 | 3.38 | 3.07 | 0.78 | 0.0 | 14.25 |
| 1.016 | 15.20 | 4.87 | 3.80 | 0.0 | 2.82 | 16.60 | 9.35 | 0.78 | 0.0 | 52.64 |
| 1.01 | 6.84 | 5.10 | 2.16 | 0.0 | 1.48 | 4.74 | 1.65 | 0.0 | 0.0 | 21.97 |
| 1.007 | 2.60 | 0.64 | 2.06 | 0.0 | 1.04 | 1.76 | 1.66 | 0.78 | 0.0 | 9.76 |
| 1.016 | 4.97 | 2.45 | 2.20 | 0.0 | 1.77 | 6.46 | 5.10 | 0.0 | 0.0 | 22.95 |
| 1.029 | 17.30 | 4.68 | 13.50 | 2.45 | 8.28 | 18.72 | 14.30 | 0.0 | 0.0 | 79.23 |
| 1.018 | 5.48 | 3.36 | 2.53 | 0.0 | 1.25 | 6.05 | 2.17 | 0.0 | 0.0 | 20.84 |
